# A Quinoline Derivative HZ-6d Induces Antiviral Activity Against Hepatitis C Virus Via Apoptosis-Mediated Cytotoxicity

**DOI:** 10.24546/0100497747

**Published:** 2025-10-03

**Authors:** GEDE NGURAH RSI SUWARDANA, AULIA FITRI RHAMADIANTI, TAKAYUKI ABE, LIN DENG, CHIEKO MATSUI, MOTOHIRO YASUI, NORIHIKO TAKEDA, TAKAHIRO YAMADA, MASAFUMI UEDA, IKUO SHOJI

**Affiliations:** 1 Division of Infectious Disease Control, Center for Infectious Diseases, Kobe University Graduate School of Medicine, Kobe, Japan; 2 Department of Microbiology, Faculty of Medicine, Udayana University, Bali, Indonesia; 3 Department of Virology, Niigata University Graduate School of Medical and Dental Sciences, Niigata, Japan; 4 Medicinal Chemistry Laboratory, Kobe Pharmaceutical University, Kobe, Japan

**Keywords:** HBV, HCV, Quinoline derivative, Anti-viral activity, Apoptosis

## Abstract

We previously demonstrated that interferon-stimulated gene 15 protein (ISG15) plays a role in enhancing hepatitis B virus (HBV) and hepatitis C virus (HCV) infections through the ISGylation of the HBV X protein (HBx) and the HCV NS5A protein. ISGylation is a post-translational modification where ISG15 is covalently attached to target proteins. These findings suggest that targeting ISGylation could be a potential therapeutic strategy for HBV and HCV infections. In this study, we evaluated the antiviral activity of HZ-6d, a quinoline derivative that inhibits HERC5-mediated ISGylation. Our results showed that HZ-6d did not inhibit HBx ISGylation and only modestly suppressed HBV replication in HBV-infected HepG2-NTCP cells and in HBV-replicating cells. Interestingly, HZ-6d also did not affect NS5A-ISGylation, however, it significantly suppressed HCV replication. These observations suggest that HZ-6d exerts its antiviral effects in HCV-replicating cells through mechanisms independent of HERC5-mediated NS5A-ISGylation. Furthermore, HZ-6d strongly activated the p53-mediated apoptosis signaling pathway, as evidenced by increased levels of phosphorylated p53, cleaved caspase-8, and cleaved caspase-3. Notably, activation of caspase-3 has been implicated in the proteolysis of HCV NS5A, indicating that apoptosis-related mechanisms may contribute to HCV suppression. Collectively, our findings suggest that HZ-6d induces antiviral activity against HCV through the p53-mediated apoptosis pathway, rather than by interfering with HERC5-mediated NS5A ISGylation.

## INTRODUCTION

Chronic infections with hepatitis B virus (HBV) or hepatitis C virus (HCV), both of which are major contributors to the global burden of hepatocellular carcinoma (HCC), accounting for approximately 56% and 20% of cases, respectively (1–3). The primary goals of therapy for HBV or HCV infections are to prevent cirrhosis, hepatic decompensation, HCC, and related mortality. Reducing the risk of HCC depends on maintaining sustained virological remission. Current treatment options for chronic HBV infection include pegylated interferon-α (Peg-IFNα) and nucleot(s)ide analogs, while direct-acting antivirals (DAAs) are the standard of care for HCV infection (4, 5). However, DAA resistance can occur in some patients, highlighting the need for alternative or adjunct therapeutic strategies.

ISG15 is the ubiquitin-like protein induced by type I interferon stimulation or by viral and bacterial infections (6). ISG15 is covalently attached to lysine residues on target proteins through a cascade involving E1 activating enzyme (UBE1L), E2 conjugating enzyme (UbcH8), and E3 ligase (HERC5). This post-translational modification, known as ISGylation, is analogous to ubiquitination and other ubiquitin-like pathways, such as SUMOylation (7). HERC5, a HECT-type E3 ligase, mediates ISGylation of numerous cellular and viral proteins (8). ISG15 can be removed from its substrates by the deconjugating enzyme USP18 (also referred to as UBP43), which cleaves isopeptide bond between ISG15 and the substrate (9).

Quinoline and quinazoline derivatives are heterocyclic compounds composed of fused aromatic rings containing nitrogen atoms. These compounds exhibits diverse biological activities, including anti-cancer, antiviral, anti-inflammatory, and antibacterial effects (10, 11). Notably, Wang et al. reported that a novel quinoline derivative, HZ-6d, inhibits HERC5-mediated ISGylation of p53, preventing its proteasomal degradation and thereby suppressing tumorigenesis via p53-mediated apoptosis (12).

Our previous studies demonstrated that HBx and HCV NS5A are substrates of HERC5-mediated ISGylation, and that this modification enhances HBV and HCV replication, respectively, functioning as a proviral mechanism (13, 14). These findings suggest that targeting HERC5-mediated ISGylation could offer a novel therapeutic approach to limit HBV and HCV replication.

In the present study, we investigated the antiviral activity of HZ-6d against HBV and HCV infections and explored its mechanism of action. We found that HZ-6d did not inhibit the ISGylation of HBx or HCV NS5A. However, HZ-6d strongly activated p53-mediated apoptotic signaling, as evidenced by increasing phosphorylation of p53 and cleavage of caspase-3. These findings suggest that the antiviral activity of HZ-6d may be attributed to apoptosis induction rather than inhibiting ISGylation.

## MATERIALS AND METHODS

### Cell culture and viruses

Huh7.5 cells were kindly provided by Dr. C.M. Rice (The Rockefeller University, NY, USA). Huh7.5 and HepG2 cells were cultured in high-glucose Dulbecco’s modified Eagle’s medium (DMEM) containing L-glutamine (Fujifilm Wako Pure Chemical Industries, Osaka, Japan), supplemented with 100 IU/ml penicillin, 100 μg/ml streptomycin (Gibco, Grand Island, NY, USA), and 10% heat-inactivated fetal bovine serum (FBS) (Biowest, Nuaillé, France), and maintained at 37 in a 5% CO_2_ incubator. Doxycycline (DOX)-inducible HBV-expressing Hep38.7-Tet cells (a gift from Dr. K. Watashi, NIID, Tokyo, Japan) were maintained in DMEM/F-12 (Gibco) supplemented with 10 mM HEPES (Gibco), 100 IU/ml penicillin, 100 μg/ml streptomycin, 10% FBS, 5 μg/mL insulin (Sigma-Aldrich, St. Louis, MO, USA), 400 μg/mL G418 (Nacalai Tesque, Kyoto, Japan), and 400 ng/ mL doxycycline.

Stable HepG2-NTCP-Myc cells were established via lentivirus transduction. The human NTCP tagged with Myc-His_6_ was amplified from the pEF1A-NTCP-Myc-His_6_ plasmid and subcloned into the pLVSIN-EF1A vector (TaKaRa Bio, Shiga, Japan). Lentiviral particles were generated using the Vesicular Stomatitis Virus-G protein (VSV-G) system by co-transfection of pLVSIN-EF1A-NTCP-Myc-His_6_ and the Lentiviral High Titer Packaging Mix (Takara Bio) into Retrovirus Constructive (G3T-hi) cells (TaKaRa Bio). Culture supernatants were collected 48 h post-transfection, filtered using a 0.45 μM membrane filter (Merck, Darmstadt, Germany), and used to infect HepG2 cells. Transduced cells were maintained in DMEM supplemented with 100 units/ml penicillin, 100 μg/ml streptomycin, 0.1 mM non-essential amino acid, 10% heat-inactivated FBS, and 1 μg/mL puromycin.

The pFL-J6/JFH1 plasmid encoding the full-length chimeric HCV-2a genome (J6/JFH1), was kindly provided by Dr. C.M. Rice. The HCV genome RNA was synthesized *in vitro* using pFL-J6/JFH1 as a template and was transfected into Huh-7.5 cells by electroporation (14). The virus particles released into the culture supernatant were used for the titration of virus infectivity. Virus infection was performed at a multiplicity of infection (MOI) of 1 in the infection experiments.

### Compounds

Compound HZ-6d was synthesized from commercial 2-hydroxy-5-methoxybenzaldehyde in 7 steps according to modified reported method (15, 16) at Kobe Pharmaceutical University. Entecavir and gefitinib were purchased from Sigma-Aldrich (Darmstadt, Germany).

### Plasmid construction

The following plasmids were used as previously described (13, 14): pEF1A-HBx-Myc-His_6_, pEF1A-NS5A-Myc His_6_, pCAG-UBE1L, pCAG-UbcH8, pCAG-HA-HERC5, and pCAG-FLAG-ISG15.

### Antibodies

The mouse monoclonal antibodies (mAbs) used in this study were anti-FLAG (M2) mAb (F-3165; Sigma-Aldrich, USA), anti-p53 (Ab-6) mAb (OP43; Sigma Aldrich), anti-HBc mAb (7B2) was kindly provided by Dr. A. Ryo (NIID, Tokyo, Japan), anti-caspase-8 (1C12) (#9746, Cell Signaling Technology) and anti-glyceraldehyde-3-phosphate dehydrogenase (GAPDH) mAb (014-25524; FUJIFILM Wako Pure Chemical Industries). The rabbit monoclonal antibodies (mAbs) used in this study were anti-cleaved caspase-3 (D175) (5A1E, Cell Signaling Technology) and anti-cleaved caspase-8 (D374) mAb (18C8, Cell Signaling Technology). The rabbit polyclonal antibodies (pAbs) used in this study were anti-HA pAb (H-6908; Sigma-Aldrich, USA), anti-HBx pAb (39716; Abcam, Cambridge, UK), anti-NS5A (2914-1) and anti-NS3 (JFH1) pAbs (kind gifts from Dr. T. Wakita, NIID, Tokyo, Japan), anti-phospho-p53 (Ser15) (#9284, Cell Signaling Technology, Beverly, MA, USA), anti-PARP (#9542; Cell Signaling Technology, Beverly, MA, USA), and anti-caspase-3 (#9662, Cell Signaling Technology). Horseradish peroxidase (HRP)-conjugated anti-mouse IgG (Cell Signaling Technology, USA) and HRP-conjugated anti-rabbit IgG (Cell Signaling Technology, USA) were used as secondary antibodies.

### HBV infection

HepG2-NTCP-Myc cells were seeded in 24-well plates at a density of 2 × 10^5^ cells/well. Cells were infected with purified HBV particles from Hep38.7-Tet cells at 1,000 genome equivalents (GEq)/cell in the presence of 4% polyethylene glycol 8000 (Hampton Research, Aliso Viejo, CA, USA) and either DMSO or 1 μM HZ-6d for 24 h. After HBV particles and compounds were washed out, the cells were cultured for 10 additional days with DMSO, HZ-6d (0.25, 1, or 4 μM), or 1 μM entecavir (ETV) as the positive control. Medium were refreshed on days 1, 4, and 7 post-infection. Supernatants and cell lysates were collected on day 10 and stored at −80°C until further use.

### HCV infection

Huh7.5 cells were incubated with HCV inoculum for 4 h at 37°C. Cells were then cultured in complete DMEM supplemented with DMSO, HZ-6d (0.3, 1, or 3 μM), or 10 μM gefitinib. Cells were harvested at 2 and 4 days post-inoculation for reverse transcribed (RT)-quantitative PCR (qPCR) and immunoblot analysis.

### Immunoprecipitation and immunoblot analysis

Cells were transfected with the plasmids using Trans IT reagents (Promega) and harvested at 48 h post-transfection. Cells were lysed in 400 μL of RIPA buffer containing 50 mM Tris-HCl (pH 8.0), 150 mM NaCl, 0.1% SDS, 1% NP-40, 0.5% deoxycholate, and protease inhibitor cocktail tablets (Roche Molecular Biochemicals, Mannheim, Germany). Cell lysates were incubated at 4°C for 2 h and centrifuged at 20,400 × g for 30 min at 4°C (TOMY centrifuge MX-307, Rotor Rack AR015-SC24; TOMY, Tokyo, Japan). The supernatants were incubated with primary antibodies at 4°C overnight and immunoprecipitated using protein G-Sepharose 4 Fast Flow (GE, Healthcare, Buckinghamshire, UK).

Samples were washed, boiled in the SDS sample buffer, and subjected to SDS-10% polyacrylamide gel electrophoresis (SDS-PAGE) and transferred to polyvinylidene difluoride membranes (PVDF) (Millipore). The membranes were blocked with Tris-buffered saline containing 20 mM Tris-HCl (pH 7.6), 135 mM NaCl, and 0.05% Tween 20 (TBST) containing 5% skim milk at room temperature for 2 h and incubated with the primary antibodies. The membranes were then incubated with HRP-conjugated secondary antibody at room temperature for 2 h. The immune complexes were visualized with ECL Western blotting detection reagents (GE Healthcare, Buckinghamshire, UK) and detected by an ImageQuant 800 system (GE Healthcare, UK).

### Cell Viability assay

The effect of HZ-6d on cell viability was evaluated using the CellTiter-Glo® 2.0 assay system (Promega, USA) according to the manufacturer’s instructions. Cells were subjected to Cell-Titer Glo analysis at 5 days after compound incubation and measured by Glomax® Navigator Microplate Luminometer GM2000 (Promega, USA).

### Real-time PCR

Total RNA was extracted using the RNeasy mini kit (Qiagen, Valencia, CA, USA), followed by cDNA synthesis using a GoScript™ reverse transcription system (Promega, USA). qPCR was performed using SYBR *Premix Ex Taq*™ II (Tli RNaseH Plus) (TaKaRa Bio, Shiga, Japan) according to the manufacturer’s instructions. Fluorescent signals were analyzed by a StepOne Plus real-time PCR system (Applied Biosystems Inc., Foster City, CA, USA). The intracellular HCV RNA, and GAPDH genes were amplified using the specific primer pairs as follows: HCV sense primer, 5′-AGACGTATTGAGGTCCATGC-3′ and HCV antisense primer, 5′-CCGCAGCGACGGTGCTGATAG-3′; GAPDH sense primer, 5′ GCCATCAATGACCCCTTCATT-3′ and GAPDH antisense primer, 5′-TCTCGCTCCTGGAAGATGG-3′. The expression level of each gene was determined by the ΔΔ*C_T_* method using GAPDH as an internal control.

The extracellular HBV DNA was extracted from cell supernatants using a QIAamp DNA mini kit (Qiagen). The HBV DNA was quantified by real-time PCR using the primer pairs 5′-GGAGGGATACATAGAGGTTCCTTGA-3′ and 5′-GTTGCCCGTTTGTCCTCTAATTC-3′ with serial dilution of 100 ng/μL plasmid pUC19-HBV-D-IND60 as the standard.

### Statistical analysis

All data are presented as the mean ± standard deviation (s.d.). Statistical analyses were performed using SPSS v28 (IBM, Chicago, IL). Depending on the normality of the data, Student’s t-test, Mann-Whitney U-test, or Kruskal-Wallis test was applied. A *p*-value < 0.05 was considered statistically significant.

## RESULTS

### Cytotoxicity profile of HZ-6d

**Figure 1:**
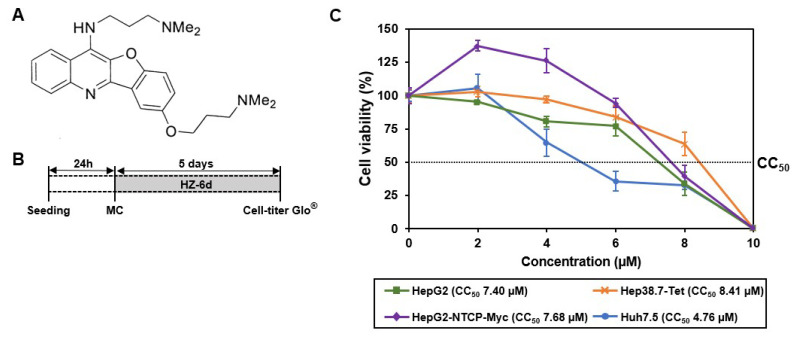
Cytotoxicity characteristics of HZ-6d **(A)** Chemical structure of quinoline derivative HZ-6d. **(B)** Schematic representation of the Cell-titer Glo v2.0 assay protocol (Promega) used to assess cell viability following compound treatment. MC: media change. **(C)** Cell viability assay results for HepG2, HepG2-NTCP-Myc, Hep38.7-Tet, and Huh7.5 cells treated with increasing concentrations (0, 2, 4, 6, 8, or 10 μM) of HZ-6d for 5 days. The half-maximal cytotoxic concentration (CC_50_) was calculated for each cell line using a dose-response curve based on the viability data. The data represent the mean ± s.d. of three independent experiments, each with n = 4 biological replicates.

HZ-6d is a quinoline derivative, with its chemical structure shown in Figure 1A. To assess its cytotoxicity, we performed a cell viability assay using three HBV-relevant cell lines (HepG2, HepG2-NTCP-Myc, and Hep38.7-Tet) and one HCV-relevant cell line (Huh7.5). Cells were treated with HZ-6d at concentrations of 2, 4, 6, 8, or 10 μM for five days (Figure 1B). The half-maximal cytotoxic concentrations (CC_50_) were calculated as 7.4 μM for HepG2, 7.68 μM for HepG2-NTCP-Myc, and 8.41μM for Hep38.7-Tet cells (Figure 1C). In contrast, Huh7.5 cells exhibited higher sensitivity with a CC_50_ of 4.76 μM. Based on these results, we selected 0.25, 1, and 4 μM of HZ-6d for HBV-related experiments, and 0.3, 1, or 3 μM for HCV-related experiments.

### HZ-6d does not inhibit ISGylation of HBx or HCV NS5A

**Figure 2:**
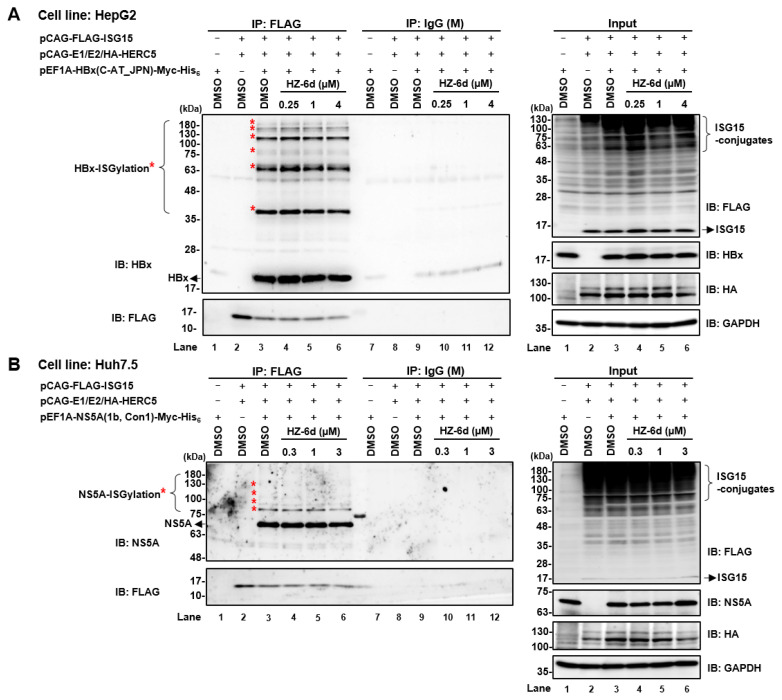
HZ-6d does not impede HBx and HCV NS5a ISGylation **(A)** HepG2 cells were transfected with plasmids pEF1A-HBx (C-AT_JPN)-Myc-His_6_, pCAG-FLAG-ISG15, and the ISGylation machinery components pCAG-UBE1L (E1), pCAG-UbcH8 (E2), and pCAG-HA-HERC5 (E3). Cells were treated with DMSO or HZ-6d for 24 h. Cell lysates were subjected to immunoprecipitation (IP) with anti-FLAG (M2) mouse monoclonal antibody (mAb), followed by immunoblotting (IB) with anti-HBx rabbit polyclonal antibody (pAb). Input controls were analyzed with anti-HBx rabbit pAb, anti-FLAG (M2) mouse mAb, anti-HA rabbit pAb, or anti-GAPDH mouse mAb. **(B)** Huh7.5 cells were co-transfected with plasmids pEF1-NS5A (1b, Con1)-Myc-His_6_, pCAG-FLAG-ISG15, pCAG-UBE1L (E1), pCAG-UbcH8 (E2), and pCAG-HA-HERC5 (E3). Cells were treated with DMSO or HZ-6d for 24 h. Cell lysates were subjected to IP with anti-FLAG (M2) mouse mAb, followed by IB with anti-NS5A rabbit pAb. Input controls were analyzed with anti-NS5A rabbit pAb, anti-FLAG (M2) mouse mAb, anti-HA rabbit pAb, or anti-GAPDH mouse mAb. The immunoblots of Figure 2A and 2B are representative images from three independent experiments that yielded similar results.

To determine whether HZ-6d interferes with ISGylation of HBx, HepG2 cells were co-transfected with plasmids encoding E1 (UBE1L), E2 (UbcH8), E3 (HA-HERC5), and FLAG-ISG15, and pEF1HBx-Myc-His_6_. Cell lysates were subjected to co-immunoprecipitation followed by immunoblotting. No signal was detected in empty vector controls (lanes 1 and 2) or in samples precipitated with mouse IgG (lanes 7–12), confirming the specificity of HBx–ISG15 interaction (Figure 2A, left panel). Comparable levels of ISGylated HBx were observed in cells treated with DMSO and with 0.25, 1, or 4 μM HZ-6d (lanes 3–6), suggesting that HZ-6d does not impair HBx–ISGylation.

Similarly, Huh7.5 cells were transfected with pEF1A-NS5A-Myc-His_6_ to evaluate HZ-6d’s effect on NS5A–ISGylation (Figure 2B). Treatment with 0.3, 1, or 3 μM HZ-6d did not significantly reduce the ISGylation levels compared to DMSO (Figure 2B, left panel, lane 3 vs. lanes 4–6). These findings indicate that HZ-6d does not interfere with HERC5-mediated ISGylation of HBx or NS5A.

### HZ-6d slightly inhibits HBV infection

**Figure 3:**
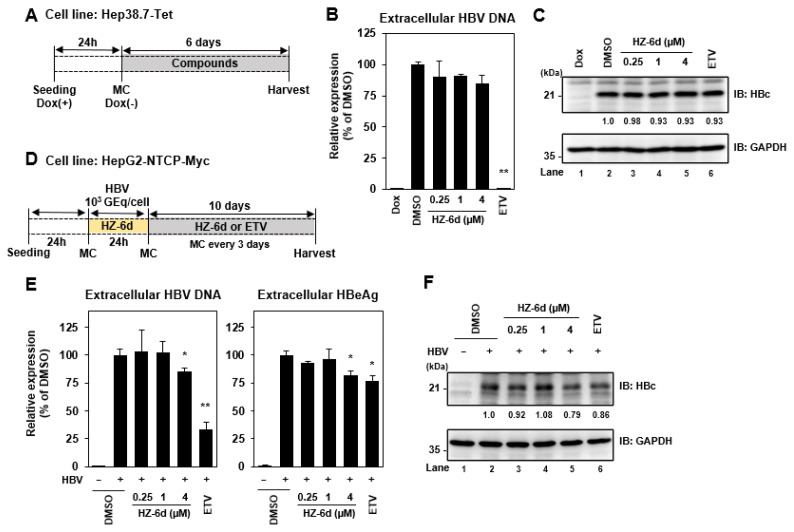
HZ-6d has a very modest suppression of HBV infection (A) Schematic overview of anti-HBV assay using Hep38.7-Tet cell system. Cells were pretreated with 500 ng/mL doxycycline (Dox) for 24 h, followed by administration of HZ-6d (0.25, 1, or 4 μM) or 1 μM entecavir (ETV) for 6 days. Supernatants and cell lysates were collected for quantification of HBV DNA by real-time PCR (B) and HBc protein analysis by western blot (C), respectively. (D) Schematic overview of HBV infection assay using HepG2- NTCP-Myc cells. Cells were infected with HBV at 1,000 genome equivalent (GEq)/cell. After 10 days of continuous treatment with HZ-6d or ETV, supernatants and cell lysates were collected for HBV DNA quantification by realtime PCR and HBeAg measurement by ELISA (E), and HBc protein analysis by western blot (F). The data in Figure 3B and 3E represent the mean ± s.d. from three independent experiments, each with n = 3 biological replicates. *p < 0.05; **p < 0.01; vs. DMSO-treated control. Densitometric analysis of the HBc band on (C) and (F) was normalized to GAPDH protein levels in each respective blot. The immunoblots of Figure 3C and 3F are representative images from three independent experiments that yielded similar results. MC: media change.

To evaluate anti-HBV activity, Hep38.7-Tet cells were treated with DMSO, HZ-6Z (0.25, 1, or 4 μM), or 1 μM entecavir in the absence of doxycycline for 6 days (Figure 3A). At 24 h after cells were seeded with doxycycline supplementation, Hep38.7-Tet cells were treated with DMSO, 0.25, 1, or 4 μM of HZ-6d, and 1 μM of entecavir as positive controls for 6 days without doxycycline (Figure 3A). As a positive control, entecavir markedly suppressed HBV replication (Figure 3B). However, HZ-6d had no effect on HBV DNA levels. Immunoblotting showed that neither HZ-6d nor entecavir suppressed HBc protein levels (Figure 3C, lane 2 vs. lanes 3–6), probably due to the tetracycline regulatory element (TRE)-regulated nature of HBV protein expression in Hep38.7-Tet cells (Figure 3C). Only doxycycline significantly suppressed HBc levels (Figure 3C, lane 1 vs. lane 2).

We also investigated the effect of HZ-6d on HBV-infected HepG2-NTCP-Myc cells. The cells were infected with HBV genotype D particles purified from Hep38.7-Tet cells and maintained for 10 days post-infection with continuous treatment of HZ-6d (0.25, 1, or 4 μM) or 1 μM entecavir, as illustrated in Figure 3D. As shown in Figure 3E (left panel), treatment with 4 μM HZ-6d led to a slight reduction in HBV DNA levels, whereas entecavir treatment resulted in a marked decrease. HBeAg was also slightly reduced by both 4 μM HZ-6d and 1 μM entecavir treatments (Figure 3E, right panel). In addition, HBc protein level was slightly reduced in cells treated with either 4 μM HZ-6d or 1 μM entecavir, compared to DMSO-treated controls (Figure 3F, lanes 5–6 vs. lane 2). Although the levels of HBeAg and HBc protein were comparable between 4 μM HZ-6d and 1 μM entecavir treatments, HBV DNA levels were significantly suppressed by entecavir. These results suggest that HZ-6d does not significantly inhibit HBV replication, while high-dose HZ-6d may slightly suppress HBV transcription.

### HZ-6d significantly inhibits HCV infection

**Figure 4:**
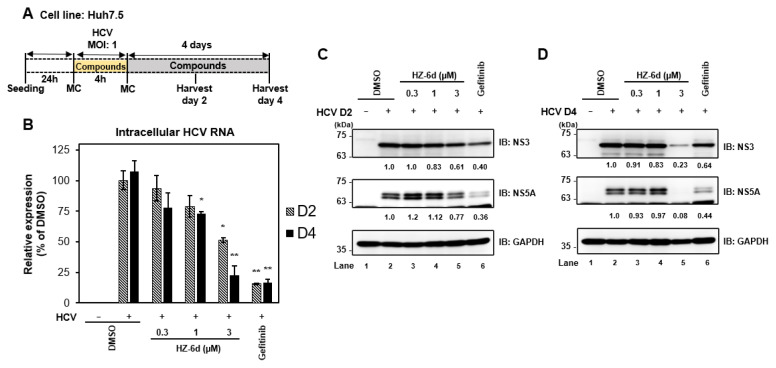
HZ-6d significantly suppresses HCV infection in Huh7.5 cells (A) Schematic overview of the anti-HCV assay. Huh7.5 were infected with HCV J6/JFH1 at a multiplicity of infection (MOI) of 1 in the presence of DMSO, HZ-6d (0.3, 1, or 1 μM), or 10 μM gefitinib. Cell lysates were collected at 2 and 4 days post-inoculation for subsequent analyses. MC: media change. (B) Quantification of intracellular HCV RNA by real-time RT-PCR at 2 and 4 days post-infection. Data were normalized to GAPDH mRNA levels. The data represent the mean ± s.d. from three independent experiments, each with n = 3 biological replicates. *p < 0.05; **p < 0.01; vs. DMSO-treated control (2 days post infection). (C, D) Immunoblotting of HCV NS3 and NS5A proteins in cell lysates harvested at 2 days (C) and 4 days (D) post-infection. Densitometric quantification of NS3 and NS5A bands was normalized to GAPDH protein levels. The immunoblots of Figure 4C and 4D are representative images from three independent experiments that yielded similar results.

To test the antiviral activity of HZ-6d against HCV, Huh7.5 cells were infected with HCV J6/JFH1 at an MOI of 1 and treated with DMSO, HZ-6d (0.3, 1, or 3 μM), or 10 μM gefitinib for 4 h (Figure 4A). Cells were harvested at 2 and 4 days post-infection. Quantitative RT-PCR showed a significant reduction in intracellular HCV RNA in cells treated with 3 μM HZ-6d or 10 μM gefitinib at both time points (Figure 4B). A modest decrease was also observed with 1 μM HZ-6d at day 4. Immunoblotting revealed that NS3 and NS5A protein levels were significantly decreased by 3 μM HZ-6d and 10 μM gefitinib at day 4 (Figures 4C and 4D). These results indicate that HZ-6d inhibits HCV replication through a mechanism distinct from ISGylation inhibition, as NS5A ISGylation was unaffected (Figure 2B).

### HZ-6d activates the p53-mediated apoptosis pathway

In Figure 4D, cells treated with 3 μM of HZ-6d at showed reduced GAPDH level compared to DMSO (Figure 4D, lower panels, lane 5 vs. lane 2), suggesting possible cytotoxic effects. To investigate whether HZ-6d triggers p53-dependent apoptosis, HepG2 cells and Huh7.5 cells were treated with increasing concentrations of HZ-6d (0.1p53-dependent apoptosis, HepG2 cells and Huh7.5 cells were treated with increasing concentrations of HZ-6d (0.1p53-dependent apoptosis, HepG2 cells and Huh7.5 cells were treated with increasing concentrations of HZ-6d (0.1

**Figure 5:**
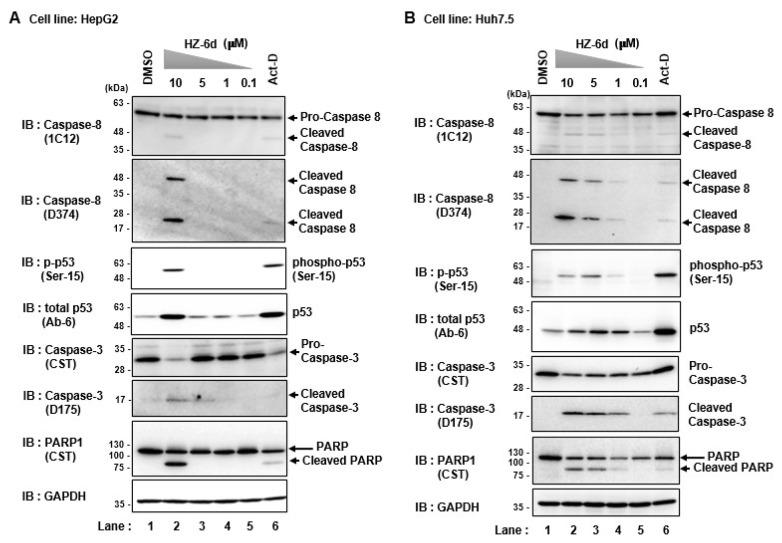
HZ-6d induces activation of the p53-mediated apoptosis signaling pathway HepG2 (A) and Huh7.5 (B) cells were treated with various concentration of HZ-6d (0.1, 1, 5, and 10 μM) or 200 ng/ml of Actinomycin-D (Act-D) as a positive control. After 18 h of incubation, cell lysates were analyzed by immunoblotting using anti-PARP1rabbit pAb, anti-p53 (Ser-15) rabbit pAb, anti-p53 mouse mAb, anti-caspase-3 rabbit pAb, anti-cleaved caspase-3 rabbit mAb, anti-caspase-8 mouse mAb, anti-cleaved caspase-8 rabbit mAb, or anti-GAPDH mouse mAb as a loading control. The immunoblots of Figure 5A and 5B are representative images from three independent experiments that yielded similar results.

In HepG2 cells, 10 μM HZ-6d induced cleavage of caspase-8, caspase-3 and PARP1 as well as phosphorylation of p53 at Ser15 (Figure 5B). The apoptosis signature resembled that induced by actinomycin D (ActD), a known activator of p53.

**Figure 6:**
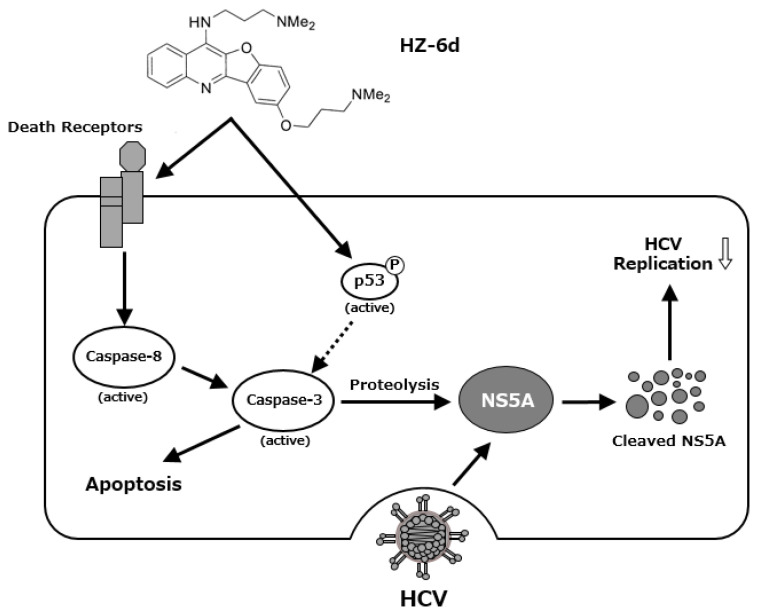
A proposed mechanism of the HZ-6d-mediated HCV suppression HZ-6d may induce the activation of caspase 8 and caspase 3 via death receptors (DRs)-mediated apoptosis pathway. HZ-6d may induce p53 activation and p53 phosphorylation, resulting in activation of caspase 3. The activated caspase-3 may cleave HCV NS5A protein, thereby suppressing HCV replication.

Moreover, in the Huh7.5 cells, administration of 1 μM HZ-6d was shown to induces apoptosis signaling pathway, especially the phosphorylation of p53, cleaved caspase 3, and cleaved PARP1 (Figure 5B). Whereas, administration of 5 and 10 μM HZ-6d on Huh7.5 cells significantly induces apoptosis signaling pathway as indicated by cleaved caspase-8, phosphorylation of p53, the cleaved caspase-3, and the cleaved PARP1. Given that caspase-3 has been reported in the proteolysis of HCV NS5A protein, we propose a model in which HZ-6d suppresses HCV replication via p53-mediated apoptosis, particularly through proteolysis of HCV NS5A protein (Figure 6).

## DISCUSSION

In this study, we demonstrated that HZ-6d, a quinoline derivative, exerts anti-HCV activity and modest anti-HBV effects. While HZ-6d was previously reported to inhibit HERC5 and reduce p53-ISGylation through binding to G-quadruplex structures in the HERC5 gene (12), our findings suggest an alternative mechanism of antiviral action. Our data clearly show that HZ-6d does not inhibit HER-5-mediated ISGylation of HBx or NS5A (Figure 2). Instead, we observed robust activation of the p53-dependent apoptosis pathway, including p53 phosphorylation and cleavage of PARP1, caspase-8, and caspase-3 (Figure 5). Caspase-3 activation is known to induce proteolysis of HCV NS5A (17, 18), potentially explaining the reduced NS5A levels and suppressed HCV replication following HZ-6d treatment. Thus, we propose that HZ-6d suppresses HCV through apoptosis induction rather than ISGylation interference.

The relevance of this apoptotic response in HBV infection remains unclear. Although HZ-6d only slightly suppressed HBV-related markers (HBV DNA, HBeAg, and HBc) at high doses (Figure 3), its overall impact on HBV replication was limited. We have to rule out the possibility that the reduced cell viability of the Huh-7.5 cells after HZ-6d treatment may have contributed to the observed suppression of HCV replication. However, our results showed a marked decrease in NS5A protein levels after treatment with 3 μM HZ-6d (Figure 4, middle panel, lane 5), while only a slight reduction in GAPDH levels was observed (Figure 4, bottom panel, lane 5). These data suggest that HZ-6d suppresses HCV replication by promoting NS5A degradation without affecting overall cell viability. We need to address this limitation in the future study and further evaluate the anti-HCV effect of HZ-6d using more physiologically relevant models, such as primary human hepatocytes.

Notably, other quinoline and quinazoline derivatives have been reported to possess antiviral effects against human cytomegalovirus (HCMV) and severe acute respiratory syndrome-2 (SARS-CoV-2), and HBV (19–21). For instance, Qiu and colleagues demonstrated that novel quinazolinone derivatives suppressed wild-type and drug-resistant HBV strains, with some compounds also inducing apoptosis in hepatoma cells (21, 22). Consistent with these findings, our study supports the potential of HZ-6d as an anti-HCV agent. However, more detailed apoptosis assays, such as terminal deoxynucleotidyl transferase dUTP nick end labeling (TUNEL) or Annexin V staining to determine the pro-apoptotic effects of HZ-6d, are needed before drawing a conclusion regarding the anti-tumor potential of HZ-6d.

In conclusion, our data suggest that HZ-6d inhibits HCV replication by inducing p53-mediated apoptosis, rather than interfering with ISGylation of viral proteins. Although its anti-HBV activity is modest, its ability to activate apoptotic signaling in hepatoma cells warrants further exploration as a therapeutic candidate for HBV- and HCV-associated liver diseases.
